# Supporting the health and well-being of school-aged children through a school nurse programme: a realist evaluation

**DOI:** 10.1186/s12913-018-3480-4

**Published:** 2018-08-28

**Authors:** Lawrence Doi, Deborah Wason, Stephen Malden, Ruth Jepson

**Affiliations:** 1 Scottish Collaboration for Public Health Research and Policy, School of Health in Social Science, Doorway 6, Old Medical School, Teviot Place, University of Edinburgh, Edinburgh, EH8 9AG UK; 2 Evaluation Team, NHS Health Scotland, Edinburgh, UK

**Keywords:** Scotland, School nurse, Pupil, Realist evaluation, Programme theory

## Abstract

**Background:**

The school nurse’s role varies across countries. In Scotland, the Chief Nursing Officer recommended that the role should be refocused. The refocused programme emphasises nine care pathways with a view to improve pupils’ health and wellbeing. Two sites were identified to test this new programme. Our aim was to assess how, for whom and under what circumstances the programme works in order to provide learning to support school nurse training and intended national roll-out.

**Methods:**

This study was a mixed methods study, using a realist evaluation approach, and conducted in three phases. In phase one, six nurse managers from both study sites took part in individual interviews or focus groups and this was complemented by programme documents to develop initial programme theory. In phase two, the programme theory was tested using qualitative data from 27 school nurses, and quantitative data from the first 6 months of the programme that captured patterns of referral. The programme theory was refined through analyses and interpretation of data in phase three.

**Results:**

The findings show that the programme enhanced opportunities for early and improved identification of health and wellbeing needs. The context of the nine pathways worked through the mechanism of streamlining referral of relevant cases to school nurses, and yielded positive outcomes by extending school nurses and thus children’s engagement with wider services. The mental health and wellbeing pathway was the most frequently used, and nurses referred complex mental health cases to more specialist mental health services, but felt less equipped to deal with low to moderate cases.

**Conclusions:**

The programme facilitated early identification of risk but was less successful at equipping school nurses to actually deliver specific interventions as intended. Capacity building strategies for school nurses should seek to enhance intervention delivery skills within the parameters of the pathways. Realist evaluation provided a useful framework in terms of identifying contextual and mechanistic influences that required strengthening prior to wider implementation.

**Electronic supplementary material:**

The online version of this article (10.1186/s12913-018-3480-4) contains supplementary material, which is available to authorized users.

## Background

School nursing is aligned with the promotion of health among school-aged children either in school or community settings. Historically, school nursing was designed as a public health measure, within the National Health Service (NHS) to address communicable diseases, inadequate nutrition in children, poor hygiene and other physical ailments that prevented children from attending school [1, 2]. The nature of school nurse’s role means they play an important part in the health and education of school children. However, evidence of effectiveness of their practice (including impact on academic performance) has been limited [1–4]. This lack of evidence of effectiveness has recently given rise to debate regarding whether the school nurse role, in its current form is still needed in today’s education system, particularly within the context of the current global economic climate. Yet if school nursing is to remain relevant it is important that the role evolves to meet the current needs of both education and health services, including the population group they serve. For example, in most countries, an increasing number of pupils are entering schools with additional and chronic health needs [5], which may require the attention of the school nurses. In order to optimally meet the needs of such children, school nurses need to ensure that their practice is based on best available evidence [6].

**Fig. 1:**
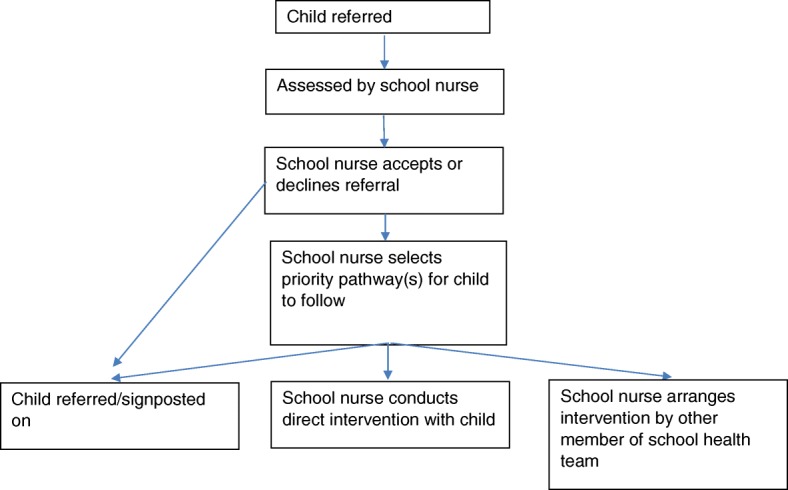
New referral pathway for school nursing

Within Scotland, the school nurse role forms part of the NHS school health service – a universally accessible service provided to children and young people, aged 5–19 years and their families. Over the years, operation of the school health service, including the school nurse role has varied greatly across Scotland. These have comprised roles and interventions focused in schools, including a remit to vaccinate all school aged children, as well as those with a wider public health and community function. In the last few years, the workload on school nurses especially in the form of extensive new immunisations increased substantially, prompting a re-consideration of the role. In 2013, the Chief Nursing Officer (CNO) of Scotland recommended the re-organisation of the school nurse role (Chief Executive Letter (CEL 13) [7]. The re-organisation was intended to refocus the role in order to deliver consistent and more efficient services across Scotland to address some of the fundamental causes of poor health and expressed need of school children and young people aged, 5–19 years by delivering safe, effective and person-centred care based on the national practice model of Getting It Right For Every Child (GIRFEC) [8]. Based on available evidence and current policy direction, the new role has been designed to ensure that there is greater emphasis on home visiting and addressing wider policy and public health priorities. As such, the new role focuses on nine priority areas or pathways: mental health and well-being, substance abuse, child protection, domestic abuse, homelessness, looked after children (children whom the state has assumed parental responsibilities), youth justice (young people involved in the justice system), young carers (children and young people who care for family members with additional needs) and transitions (children moving from one educational institute to another). School nurses will assess children and then either refer children onto the relevant services or provide direct intervention themselves (see Fig. 1 for referral pathway). As part of the NHS, school nurses have access to a child’s clinical record and will record all referrals and their outcomes into the primary care database as well as into a specially developed national core minimum dataset for school nursing.

As part of the effort to streamline the role and deliver the nine pathways, some existing duties of school nurses were assigned to existing health improvement services, or through the delivery of the health and well-being component of the school curriculum. The responsibility for immunisation of school-aged children will progressively be transferred to specialist teams. The previous and current roles of the school nurse are outlined in Table 1.

**Table 1:** Previous and current roles of the school nurse

Previous role	Current role
Responsible for immunisation of all school age children	Immunisation progressively delegated to specialist teams
Support for whole school curriculum	Individual interventions based on pupil need
Support for school children with chronic physical health conditions	Chronic physical health conditions delegated to Community Children’s Nurses
Children ‘referred’ to service via ad hoc requests e.g. being stopped in corridor by teachers	Children referred formally to service from a variety of sources including education staff, GPs, social workers etc
School nurse role ill-defined but incorporates children with almost any need	School nurses prioritise children who are referred into service on one or more of the 10 pathways.
Does not lead team	Leads a school health team possibly including health care assistants and staff grade nurses
Infrequent and ad hoc home visiting	Role includes family assessment and home visits
Limited holistic assessment of family and environment outside school	GIRFEC and wider family assessment
Unfocused and unclear contribution to outcomes such as improved mental health	Focused role with agreed definition and referral mechanisms
No nationally collected data on school nurse role	Contributes to national dataset on health of school age children

In terms of advancing the recommendation of CEL 13, the CNO commissioned a national steering group to oversee the design and testing of the refocused model in two early adopter Community Planning Partnerships (CPPs) sites - Dumfries and Galloway (Site A) and Perth and Kinross (Site B). These sites began testing the programme, including the role of the wider school health team, and associated re-design requirements from September 2015. However, in order to provide learning and guidance to support scaling up of the programme across Scotland, this study aimed to use realist evaluation to understand how the components of the contexts and mechanisms of the refocused programme influenced outcomes in both study sites. The research was funded by the Directorate for the Chief Nursing Officer, Scottish Government and the full report is available here - https://www.gov.scot/Publications/2017/07/2706/0

## Methods

### Evaluation design

A realist evaluation design was used, employing both qualitative and quantitative data. Realist evaluation is a theory-driven approach to evaluation. It involves exploration of complex interactions observed between the contexts (specific settings where the programme is implemented), mechanisms (causal forces, powers, processes or interactions that generate change within an intervention, including the choices, reasoning, and decisions that people make as a result of the resources provided by the programme), and outcomes (intended and unintended effects) involved in the programme [9–11]. Realist evaluation develops, tests and refines programme theory. As well as serving as guidance for data collection, a realist evaluation programme theory can help explain how and why a programme works, for whom, and in which contexts [9, 12]. Using the realist evaluation reporting standards [12], this evaluation proceeded in three key phases - developing, testing and refining the programme theory.

### Settings

The characteristics of the two sites are shown in Table 2.

**Table 2:** Characteristics of the study sites and schools

	Total area population	Area	Primary Schools	Secondary Schools
Site A	149,670	6426 km^2^	99	16
Site B	149,930	5286 km^2^	69	11

### Participants

Nurse managers, school nurses (including members of the wider school health team) and secondary data from school children (nursery, primary and secondary school pupils) were used in this study.

### Sampling, recruitment and data collection

#### Phase 1. Identifying the programme theory

In order to understand how assumptions underlying how the refocused school nurse programme was expected to work to achieve intended outcomes, it was necessary to collect data from the perspective of the managers involved in local planning and implementation. As such, we conducted two focus groups and three individual interviews with managers from both study sites. The focus groups examined the rationale for the programme; the assumptions about the mechanisms through which the programme was expected to work; anticipated outcomes for families; and implications for school nurse practice. Additional file 1 shows the topic guide used for the focus groups. LD and SM conducted the focus groups with six managers.

Data from the focus groups were used together with a comprehensive logic model already designed for the proposed national refocusing of the school nurse programme to develop the initial programme theories.

#### Phase 2. Testing and refining the programme theory

The programme theories were tested using two key data sources.

##### Semi-structured interviews

All school nurses and members of the wider school health team involved in the delivery of the refocused school nurse role from the two study sites were invited to participate in the study. All potential participants were sent a study information pack containing an invitation letter, information sheet and expression of interest form. Interested individuals were asked to complete and return the expression of interest form to the research team or their line manager. A member of the research team contacted potential participants directly to arrange a convenient date, time and venue for interviews.

Within Site B, all sixteen eligible school nurses and members of the wider school health team took part in the study. In Site A, all but six of the seventeen eligible school nurses and support workers participated. Overall, 27 school nurses provided data for testing the programme theories.

Interviews examined elements of the initial programme theories, with a particular focus on understanding how the mechanisms of the programme operate in practice and interact with contexts to produce intended and unintended outcomes. This was achieved by posing questions relating to practical experiences of delivering the programme. Additional file 2 shows the topic guide used for the interviews. All focus groups and interviews were audio recorded and lasted approximately 30–60 min.

##### Case record audit

In addition to the interview data, we also conducted a case record audit of all cases that referred to school nurses from November 2015 until the end of May 2016. This was done by designing a form, which school nurses were asked to complete for each new referral. The information gathered on the form included age, sex, deprivation category of child referred, reason for referral, pathway child was placed on and general information around outcomes. Although the audit data provides additional dimension to the study in terms of testing some of the initial programme theories, it does not infer a cause and effect relationship.

### Analysis

#### Phase 1

Analysis in realist evaluation is centred on the three crucial elements of contexts, mechanisms and outcomes. Using focus group data and programme documents, we produced a matrix involving these three elements and formulated them as overarching initial programme theories of the refocused school nurse programme.

#### Phase 2

This stage involved qualitative data analysis of school nurses’ interview transcripts and descriptive statistics of case record audit using SPSS to test the initial programme theories produced in phase 1.

Regarding the qualitative data analysis, we used a thematic analysis approach and relied on the three core concepts – contexts, mechanisms, and outcomes - of realist evaluation to drive the process. Two members of the research team (LD and SM) independently read and coded each transcript. Through several iterations and revisions, similar codes were grouped under overarching themes. Data were then summarised and synthesised by seeking evidence to support or refute the initial programme theories through the interaction of mechanisms and contexts of the programme components and the outcomes they produced. QSR NVivo 10 [13] facilitated the analysis.

#### Phase 3

The findings of phases 2 were used to revise the initial programme theory. The focus of the analysis at this stage was to synthesis the emerging findings in order to generate explanations about how the programme theories unfolded or did not unfold in practice, whilst identifying alternative explanations. This resulted in the generation of refined CMO configurations.

## Results

### Initial programme theory

The four initial programme theories that were identified from nurse managers’ data, and subsequently tested were:

- The nine pathways (C) lead to streamlining of referrals (M), which could improve children’s outcomes, especially for those who need the service the most (O).
- Standardisation of service and clarity of role (C) adds credibility to the school nursing role (M), and could result in enhanced professional status (O) and promote interagency working (O).
- Regarding engagement and accessibility of the school nursing role (C), opportunities to be more accessible to the wider school population have reduced, as has the perceived visibility of school nurses within school settings (M) but engagement with partner agencies and ‘high risk’ children has improved, which is important in terms of building trusting relationships (O).
- Training and support (C) could facilitate the adoption of the programme and would provide opportunity for role development (M), which would empower nurses to deliver, identify and provide appropriate support within the pathways (O).

### Testing the programme theory

#### Characteristics of cases referred to school nurses

Table 3 provides an overview of the demographic characteristics of cases referred to school nurses from November 2015 to May 2016.

**Table 3:** Demographics of children referred to school nurses

Item	Description	Site A (*n* = 299)	Site B (*n* = 107)
Gender (% of cases)	Boys	36.3	46.7
	Girls	63.7	53.3
School level (% of cases)	Nursery	0	2
	Primary	29	58
	Secondary	72	38
Deprivation^a^ (Scottish Index of Multiple deprivation (SIMD) (% of cases)	SIMD 1 (most deprived)	26	11
	SIMD 2	21	23
	SIMD 3	34	19
	SIMD 4	16	35
	SIMD 5 (least deprived)	3	12

^a^No postcode recorded for 84 pupils in site A and 6 pupils in site B

#### Components

We compared and contrasted how the programme unfolded in practice both within and between the two study sites under four components as per the initial programme theory identified through the programme documents and nurse managers’ data.

##### Component 1 – Referral system and nine pathways

This component tested how the nine pathways, which are the key aspects of the programme, were rolled out and how school nurses viewed the referral system. It was intended that some previous duties of school nurses would be discontinued in order to allow them to focus on delivering the nine pathways. Whereas this was the case in Site A, Site B continued immunisation in addition to delivering the new programme. It was unsurprising that adaptation to this major change varied between the two sites.

*“You’ve got a team of school nurses here, who are hugely experienced, good at their job, and we all felt that we just weren’t giving it enough time, and enough, you know, effort. Because we just couldn’t, because, since October, we’ve basically been immunising, from October to June” (PK3, Site B).*

Nurses from both study sites mentioned that the introduction of a more formal referral system has empowered school nurses and made their work more focused. Specifically, a number of nurses stated that the referral system encourages education staff to think more carefully about referring a child to the school nurse, as they were required to use the referral form to justify their reasons for doing so. The referral system also allowed the school nurses to review each individual case before accepting it. This also allowed them to refuse or pass on specific cases to more appropriate agencies.

*“I think the referral process is really good, because it gives the education staff a clearer focus on the children that we should be working with, rather than just a wee word in the corridor as you pass, which is what happened previously. I think the referral process is really good for education and for us as well, because we can have a much more, almost like a streamlined caseload that, you know, we’re working with children that really need to be worked with” (PK4, Site B).*

Some nurses were concerned that the pathways were numerous and quite complex, making them a little bit cumbersome to use in practice.

*“To me it’s too big, there are too many priority areas, you know, it needs to be more defined, maybe more structured. It’s a bit wordy as well, there is quite lot in it, there’s quite a lot in it, you know” (D5, Site A).*

Despite the concerns regarding the high number of pathways, some nurses struggled to fit in some conditions that were less explicit on the pathways.

*“I squeeze in children that are quite overweight and obviously need that managed and you can say it will affect their self-esteem and their confidence so you can fit it under the mental health and wellbeing pathway but actually you’re not recognising the problem” (PK7, Site B).*

Another area that divided opinion amongst nurses was the apparent omission of sexual health. Some nurses believed sexual health should be a stand-alone pathway, while others contested that it is sufficiently covered by other agencies and that there are ways of working sexual health referrals into the existing nine pathways.

*“Do you know, I just think it’s crazy that sexual health isn’t one on its own” (D9, Site A).*

Nurses at both sites stated that the pathway that presents in referrals most frequently was mental health and wellbeing. School nurses felt that the mental health and well-being pathway was sometimes used as a ‘catch all’ for occasions when an appropriate pathway was difficult to identify. In spite of this, they believed that mental health issues are increasing in children and schools see this as a key part of the school nurse’s role.

This is congruent with the case record audit, which showed that the majority of children (68%) were referred in to the programme for mental health and well-being issues as shown in Table 4.

**Table 4:** Percent of children on pathways at referral (from November 2015 to May 2016)

	Site A (%)^a^	Site B (%)^a^
Mental Health and Well-Being	68	68
Substance Misuse	0.3	0
Child Protection	4	0
Domestic Abuse	2	3
Looked After Children	12	0
Homelessness	1	5
Youth Justice	0	3
Young Carers	0.3	5
Transitions	4	0
Unknown/Discharged	9	32

^a^Children could be on more than one pathway

Nurses recognised that mental health and wellbeing was an important pathway, however a number of nurses, felt they were inadequately trained to deal with low to moderate mental health issues. While it was generally accepted that more mental health training is needed, nurses were also aware that they could refer more severe cases on to child and adolescent mental health services (CAMHS).

*“For those of us who are not mental health trained we noticed a real gap in our training there and we sort of passed that on to relevant people, but more and more the children that were coming to see us and that were asking for our help were falling into that pathway and that was an area where we all felt we lacked somewhat” (PK16, Site B).*

##### Component 2 - role clarity and standardisation

This component explored the impact of the standardised programme on the school nurse role. It appeared that within both study sites, working with agencies such as social work, sexual health and education has always been good. However, nurses mentioned that the scope of the programme has made them more aware of the other agencies they had not previously engaged with, for example youth justice.

The introduction of a referral system was generally perceived to have formalised procedures, and helped clarify the role of the school nurse to other agencies.

*“In the past people in a community, other professionals were never quite sure what we’ve done and it’s always been a, you know, yes, we’ve been needed and appreciated but I think we’ve been appreciated more, especially now we have got the referral form, it can show that, you know, we’ve got proof that we are getting referred and why they are getting referred and I think our profile has been greatly raised with the pilot” (D1, Site A).*

Whilst the role of school nurses was well defined in the programme, some members of the wider school heath team were uncertain regarding the expectations of their role. Some of them were unsettled by the inconsistencies across different areas regarding their role within the programme.

In terms of standardisation of practice, immunisation has been the most conspicuous and prevalent challenge in site B. Although site B had stopped a number of previous duties, nurses, particularly those in the lower bands were still involved in immunisation and this prevented them from engaging fully with the programme.

*“We’ve dropped a lot, we don’t do health promotion and things like that anymore, but it’s been taken up, the time that we gained by not doing that has been taken up with immunisations…I’ve not been given the opportunity to take on any of this (pilot)” (PK10, Site B).*

##### Component 3: Engagement and accessibility

This component examined how the refocused programme has influenced school nurses engagement with school children, education staff and partner agencies. Discontinuation of previous duties, for instance health promotion sessions, has reduced the visibility and accessibility of nurses to children. Many of the nurses believed that although they were not widely accessible to the wider school population, there was opportunity to build and strengthen trusting relationships with the limited children and families who accessed the programme.

*“I would say that it definitely strengthens relationships with children and families because we’ve got more focus on what we are doing” (D1, Site A).*

Some other nurses explained that this was possible because they tended to spend more time with a limited group of children and families, often involving home visits. Another study conducted with school children in both study sites suggests that children and young people believe that it is important to build trusting relationships prior to discussing sensitive issues with school nurses [14].

Previously, children could access school nurses through a drop-in service. As part of the programme, children were meant to access the services of the school nurse through pupil support teachers. However, nurses asserted that accessing the school nurse through the pupil support teachers was potentially a barrier for some children.

*“Well, when they had the drop-in they didn’t have to speak to anybody. They could have just dropped in confidentially. Now it’s not a confidential service because you’d have to go to pupil support and what happens is they may go to pupil support and say I’d quite like to see the school nurse when she’s in and pupil support may say, oh, what’s wrong, can I help at all and in the right way but that’s not...that means that you’re taking something away from that service because it’s not then as accessible as a confidential service” (D8, Site A).*

In terms of engagement with other agencies, it was clear that within site A the programme has significantly facilitated this. Conversely, it appeared that immunisation has hindered this to a large extent within site B.

It was suggested by managers that the refocused SN role would increase home visits or referrals. However, the school was the main source of referral, particularly in site B but Social Work, other health services and other agencies also referred. Most of the initial contact was made in school although the place of initial contact was often not recorded and so it is not possible to state definitively if, for instance, home visits were increasing.

##### Component 4 training to deliver pathways

This component explored how school nurses viewed the training opportunities offered by the programme and whether they felt equipped to deliver the pathways. It was consistently clear that all nurses in both study sites, especially the higher bands (with specialist practitioner qualification), received extensive training on the pathways, including those delivered by specialist mental health agencies, for example CAMHS. The training equipped nurses with the necessary skills and knowledge to facilitate the early identification of risk.

*“…and with the training we’re able to maybe identify the kind of early indicators of risk within maybe if it’s risk-taking behaviours or if it’s potential issues at home, we’re better” (D3, Site A).*

Although most nurses, regardless of their grade, found the training useful, a few thought it was quite theoretical and did not equip them with sufficient practical tools or skills to actually deliver relevant interventions. Nurses were especially keen to be up-skilled in intervention techniques around child and adolescent mental health and well-being and the various other pathways. For instance, one school nurse revealed below that whilst it is straightforward to assess risks and assign a pathway, they often lack the skills to provide appropriate intervention.

*“You’ve got the skills on maybe assessing anxiety or assessing self-harm, but what can we use to try and do a bit of work with that person? We don’t have the resources to actually implement the work there. We’ve got the knowledge of what maybe the risk factors and things are but we’ve got nothing to make any interventions with” (D3, Site A).*

Some nurses suggested that ongoing training especially on the pathways they sparsely engage with would be useful. In particular, youth justice and homelessness pathways were mentioned. Similarly, others were of the view that further training and support was required within the mental health and well-being pathway, which appeared to be the most commonly used pathway in both study sites. Nurses explained that whilst severe mental health cases are easy to refer on, they struggle to cope with low-level mental health issues.

*“I think it is when the young people or children’s come to us, and it’s a mental health issue they’ve got, I feel confident enough to know if I need to move it on quickly. Because I can recognise that, you know, if they are in a stage where I have to move it onto my mental health colleagues quickly I know that. But it’s with the ones who are just a wee bit, you know, sort of a wee bit of anxiety, a wee bit of they are feeling a bit low mood. It’s just to have more support on, you know, where we are taking them” (PK12, Site B).*

Further analysis showed that there was a need for further training on mental health and well-being. Interestingly, training needs appeared to differ disproportionately across the study sites. More nurses in site B than site A felt there was a training gap. It was also mentioned that the mental health services in site B have a long waiting time and this seemed to have necessitated the perceived training need.

It was apparent that both study sites had issues with how training would affect their existing staff capacity. There were concerns that the training opportunity offered to staff to acquire a more specialist qualification in school nursing put further pressure on the capacity of the existing workforce.

It appears that there was no noticeable difference in terms of how SN with or without SPQ felt equipped to deliver the pathways. Any difference was possibly masked by the extensive, and often mandatory training given to all SN on each of the pathways.

### Refined programme theory

The evidence from phase two suggests that the initial programme theory can be refined to enhance understanding of how the refocused programme worked. For instance, the nine pathways (context) promoted a referral system, which offered school nurses the opportunity to streamline referrals (mechanism) this led to improved identification of children’s needs, especially for children who were in need of care (outcome). However, when selecting a pathway for a child to follow (context), the mental health and well-being pathway was considered the most frequently used pathway, partly due to the broad spectrum of referrals that can be categorised as mental health-related (mechanism), however school nurses were less equipped to deal appropriately with the many referrals on this pathway (outcome).

Regarding role clarity and engagement with wider services (context), although there were uncertainties over the role of lower band staff, the pathways added more credibility to the role of the school nurse and extended the scope of partnership working (mechanism) which facilitated early identification of concerns and provision of appropriate interventions (outcome). School nurses received extensive training to deliver the pathways (context), however low engagement with pathways such as youth justice and homelessness (mechanism) led to low confidence to engage with these pathways and facilitated a perceived need for ongoing training (outcome).

## Discussion

In this realist evaluation we found that the nine pathways streamlined referral of cases and undoubtedly made the school nurse role more focused and standardised. The pathways provided a platform for early and improved identification of pupils’ health and well-being needs along specific pathways. This culminated in coordinating care and support for pupils with wider community services. Although the mental health and well-being pathway was the most frequently used, nurses referred complex mental health cases to a specialist agency, and felt less equipped to deal with low to moderate cases.

Mental health problems are highly prevalent in children and young people and globally it is estimated that 10–20% of children and adolescents are affected [15, 16]. A recent study indicates that 24% of girls and 9% of boys at age 14 years are depressed in the UK [17]. It appears that the prevalence of mental health problems in young people has decreased slightly in Scotland. Using the Warwick-Edinburgh Mental Wellbeing Scale**,** a recent Survey found that the average score for 13 and 15 year olds decreased slightly between 2010 and 2013 from 50.0 to 48.7. There has also been a minor decrease in score to 48.4 between 2013 and 2015 [18].

Addressing mental health issues, for example in schools can improve the quality of mental health care for children and young people. A study conducted in Scotland showed that psychological, emotional or behavioural issues were the most common problems school nurses encounter in their practice [19]. Similar to our current study, this previous study also found that school nurses felt less equipped to deal with low to moderate mental health cases. Unfortunately, school teachers also seem unequipped to tackle this issues in children and young people [20]. A survey in England found that over 50% of primary school teachers felt inadequately trained to support pupils with mental health problems [21]. It is apparent that more school children in the UK and perhaps globally are not receiving the needed support. As there are currently no nationally agreed guidelines on the assessment and treatment of mental health issues in young people across Scotland, it is difficult to know the most appropriate training for school nurses. However, with child and adolescent mental health services under intense pressure [22], school nurses are increasingly expected to play an important role in identifying, and perhaps providing appropriate intervention to children who are at risk of mental ill-health. It is therefore imperative to address this training gap in any nationwide implementation in order to maximise the benefits of the refocused programme.

The provision of health services in school can reduce barriers to accessing timely healthcare and school nurses are frontline staff meant to facilitate equitable access to care and coordinate care for pupils with wider community health services [23, 24]. However, in the current study we found that the frontline role of the school nurse was replaced by the practice of pupils accessing the school nursing service through the pupil support teacher. This was viewed as an important barrier and has potential implications for increasing health inequalities as less articulate pupils, perhaps with sensitive issues are less likely to access the service. As such, if the service is to facilitate equitable access to care and help promote early identification of needs, the current approach may need to be reviewed and alternative strategies that provide more equitable access to the service explored.

The use of a realist evaluation approach meant this study was not designed to provide a verdict of whether the refocused programme worked or not as characterised by traditional evaluation approaches, which often oversimplify both contexts and interventions [25, 26]. Similar to other realist evaluations, this study was keen to find out how the outcomes of the refocused programme were enabled and constrained by the interaction of the contexts and mechanisms of change [10, 27, 28]. If an evaluation design that just measure intended change was used, it would have been difficult to unearth and explain important elements regarding how the programme unravelled and the nuanced components that may require strengthening prior to a wider adoption and implementation.

### Limitations

When interpreting the findings, it is important to bear in mind that these two study sites are relatively affluent areas compared with other areas of Scotland. Also, a few school nurses declined to take part in the interviews and their reasons were not evident. Yet it is likely that they might have had strong perspective of the programme, which could have influenced the findings of this study.

### Implications for school nurse practice

It was intended that the programme would enhance school nurse’s ability to identify the needs of pupils early and address a substantial proportion of these needs by delivering specific interventions. However, school nurses felt less equipped to deliver such interventions, particularly on the mental health and well-being pathway. Although extensive training was provided to school nurses prior to implementation of the programme, it appears that school nurses would benefit from further training approaches that seek to build practical skills within the parameters of the pathways. This would ensure that aside from identifying risks, nurses would also be equipped with skills to deliver appropriate health and well-being interventions.

Although one of the aims of the programme was to standardise the service in terms of data management, we found that there were disparities in the system and format used by the two study sites. Agreement on format and consistency of data gathering would be useful in any future evaluation.

## Conclusions

The refocused school nurse programme appeared to have facilitated early identification of risks but was less successful at adequately preparing school nurses to actually deliver certain important interventions as intended. The realist evaluation approach was instrumental in terms of identifying which contextual and mechanistic influences of the programme, such as school-based mental health practices that may require strengthening prior to wider implementation of the programme across Scotland.

## Additional files

Additional file 1: Topic guide used for managers’ focus groups. (DOCX 116 kb)

Additional file 2: Topic guide used for school nurses’ interviews. (DOCX 114 kb)

## Abbreviations

CAMHS: Child and Adolescent Mental Health Services
CEL: Chief Executive Letter
CMO: Context Mechanism Outcome
CNO: Chief Nursing Officer
D & G: Dumfries and Galloway
GIRFEC: Getting It Right For Every Child
P & K: Perth and Kinross
SN: School Nurse
SPQ: Specialist Practitioner Qualification
SPSS: Statistical Package for the Social Sciences

## References

[1] Maughan E (2003). The impact of school nursing on school performance: a research synthesis. J Sch Nurs.

[2] Williams S, Dickinson A. The provision of nurse-led school-based health services. Contemp Nurse. 2017; 10.1080/10376178.2017.1350587. 10.1080/10376178.2017.1350587 28673195

[3] Wainwright P, Thomas J, Jones M (2000). Health promotion and the role of the school nurse: a systematic review. J Adv Nurs.

[4] Lee RLT (2011). The role of school nurses in delivering accessible health services for primary and secondary school students in Hong Kong. J Clin Nurs.

[5] Canter KS, Roberts MC (2012). A systematic and quantitative review of interventions to facilitate school reentry for children with chronic health conditions. J Pediatr Psychol.

[6] Adams S, McCarthy AM (2005). Evidence-based practice and school nursing. J Sch Nurs.

[7] CEL 13. Public Health Nursing Services – Future Focus. 2013. http://www.sehd.scot.nhs.uk/mels/CEL2013_13.pdf. Accessed: 14 Oct 2017.

[8] Scottish Government. A guide to getting it right for every child. 2012. http://www.gov.scot/resource/0042/00423979.pdf. Accessed 14 Oct 2017.

[9] Pawson R, Tilley N (1997). Realistic evaluation.

[10] Greenhalgh T, Humphery C, Hughes J, Macfarlane F, Butler C, Pawson R (2009). How do you modernize a health service? A realist evaluation of whole-scale transformation in London. The Milbank Quarterly.

[11] Byng R (2011). What makes a realist evaluation?. Fam Med.

[12] Wong G, Westhorp G, Manzano A, Greenhalgh J, Jagosh J, Greenhalgh T (2016). RAMESES II reporting standards for realist evaluations. BMC Med.

[13] QSR International. NVivo qualitative data analysis software. Version 9; 2015.

[14] Woodhouse A, Bainbridge J, Miller J, Thomson C, Gray H. School nursing service review: consultation with children and young people. Children in Scotland 2016. https://childreninscotland.org.uk/wp-content/uploads/2017/09/Report_School_NursesJuly_2016.pdf. Accessed 14 Oct 2017.

[15] Kieling C, Baker-Henningham H, Belfer M, Conti G, Ertem I, Omigbodun O (2011). Child and adolescent mental health worldwide: evidence for action. Lancet.

[16] Schulte-Körne G (2016). Mental health problems in a school setting in children and adolescents. Dtsch Arztebl Int.

[17] Patalay P, Fitzsimons E (2017). Mental ill-health among children of the new century: trends across childhood with a focus on age 14.

[18] Scottish Government. Scottish Schools Adolescent Lifestyle and Substance Use Survey (SALSUS) 2015: Mental Wellbeing Report. 2017. http://www.gov.scot/Resource/0051/00518032.pdf. Accessed 08 Nov 2017.

[19] Wilson P, Furnivall J, Barbour RS, Connelly G, Bryce G, Stellard A (2008). The work of health visitors and school nurses with children with psychological and behavioural problems. J Adv Nurs.

[20] Reinke WM, Stormont M, Herman KC, Puri R, Goel N (2011). Supporting children's mental health in schools: teacher perceptions of needs, roles, and barriers. Sch Psychol Q.

[21] AFNCCF. Teachers identify training gap in mental health. 2011. http://www.annafreud.org/insights/news/2017/09/teachers-identify-training-gap-in-mental-health/. Accessed 14 Oct 2017.

[22] Frith E. Centre forum commission on children and young People’s mental health: state of the nation. 2016.

[23] Parasuraman SR, Leiyu S (2015). Differences in access to care among students using school –based health centres. J Sch Nurs.

[24] Turner G, Mackay S (2015). The impact of school nurse interventions: behaviour change and mental health. Br J School Nurs.

[25] Salter KL, Kothari A (2014). Using realist evaluation to open the black box of knowledge translation: a state-of-the-art review. Implement Sci.

[26] Pfadenhauer LM, Gerhardus A, Mozygemba K, Lysdahl KB, Booth A, Hofmann B (2017). Making sense of complexity in context and implementation: the context and implementation of complex interventions (CICI) framework. Implement Sci.

[27] Wand T, White K, Patching J (2011). Realistic evaluation of an emergency department-based mental health nurse practitioner outpatient service in Australia. Nurs Health Sci.

[28] Doi L, Jepson R, Hardie S. Realist evaluation of an enhanced health visiting programme. PLoS One. 2017;2(7):–e0180569. 10.1371/journal.pone.0180569 10.1371/journal.pone.0180569 PMC5495393 28672013

