# Supplementary material for: Supporting the health and well-being of school-aged children through a school nurse programme: a realist evaluation
Source: BMC Health Serv Res. 2018 Aug 28;18:664. doi: 10.1186/s12913-018-3480-4 (PMC6114697; doi:10.1186/s12913-018-3480-4)
Supplement: Supplementary file 1 — Topic guide used for managers’ focus groups. (DOCX 116 kb) [file 12913_2018_3480_MOESM1_ESM.docx]

**Additional file 1. Topic guide used for managers’ focus groups**

- What are the key changes that have been introduced to the school nursing role?
- What do you think was the rationale for implementing the priority areas/pathways?
- What strategies or activities were put in place before the priority areas were introduced? (prompt to find out more about CPD and details about the training programme)
- Could you tell me how the priority areas were implemented?
- How is the refocused school nursing role incorporating individual or community assets and strength-based way of working?
- What specific plans/structures have been put in place to ensure that school nurses improve their knowledge and awareness of community assets and referral pathways?
- In what ways are you ensuring that school nurses are visible and accessible to school children, young people, their families and partner agencies?
- In what ways are school nurses contributing to multiagency support for keeping children safe?
- In what ways are you equipping school nurses to identify risks in children, young people and their families early and provide appropriate support?
- How was it envisaged that the changes would make things better for:

School nurses and the wider school nursing team?

Children and families?

- What do you think are the gaps in school nursing education and how can this be addressed?
- In your opinion, what are the key benefits of this refocused school nursing role?
- What were you expecting to achieve in the short, medium and long term?
- In your opinion, what have been the key challenges of implementing the priority areas?
- What might need to be in place to improve the school nursing role further?
