# Supplementary material for: Supporting the health and well-being of school-aged children through a school nurse programme: a realist evaluation
Source: BMC Health Serv Res. 2018 Aug 28;18:664. doi: 10.1186/s12913-018-3480-4 (PMC6114697; doi:10.1186/s12913-018-3480-4)
Supplement: Supplementary file 2 — Topic guide used for school nurses’ interviews. (DOCX 114 kb) [file 12913_2018_3480_MOESM2_ESM.docx]

**Additional file 2. Topic guide used for school nurses’ interviews**

- How are you responding to the refocusing of the school nurse programme?
- What do you think are now the key expectations of the school nurse programme?
- Do you feel you require additional support in this new role? (prompt to find out if there are gaps in education)
- How do you feel you are equipped to identify risks in children, young people and their families early and provide appropriate support?
- What are your opinions about the selected nine priority areas/pathways of intervention?
- In your view, which of the priority areas are more difficult to focus on and why?
- What difference do you think the changes introduced to the school nurse programme is making for:
  - Children and young people
  - Their families
  - Professional partnership/multiagency working
  - You as a school nurse
- In what ways are you ensuring that school nurses are visible and accessible to school children, young people, their families and partner agencies?
- How do you feel you are contributing to multiagency support for keeping children safe?
- Has the changes enabled you to link to wider services such as social work and sexual health in ways that you had not previously done?
- Can you give me some examples of additional interventions or supports that you have been able to access for children, young people and families due to this new way of working?
- Regarding looked after children, do you think other partner agencies have understanding of the contribution that school nurses make to the assessment process and child’s plan?
- Does the refocusing of the school nurse programme allow you to:
  - Strengthen relationships with children, young people and their families? If so how?
- In your opinion, what have been the key challenges of delivering this refocused school nurse programme?
- I understand that you have now moved to strength-based and inequalities sensitive way of working using improvement methodology.
- How do you understand the term strengths based working?
- How has this influenced your practice?
- What is your experience of working with families in this way?
